# **^1^H** NMR Spectroscopy-Based Metabolomic Assessment of Uremic Toxicity, with Toxicological Outcomes, in Male Rats Following an Acute, Mid-Life Insult from Ochratoxin A

**DOI:** 10.3390/toxins3060504

**Published:** 2011-05-26

**Authors:** Peter G. Mantle, Andrew W. Nicholls, John P. Shockcor

**Affiliations:** 1 Centre for Environmental Policy, Imperial College London, London, SW7 2AZ, UK; 2 Investigative Preclinical Toxicology, GlaxoSmithKline R&D, Park Road, Ware, Herts, SG12 0DP, UK; Email: andrew.w.nicholls@gsk.com; 3 Waters Corporation, Milford, MA 01757, USA; Email: jshockcor@me.com

**Keywords:** metabolomics, polyuria, ochratoxin A, pharmacokinetics, toxicology, nephropathy

## Abstract

Overt response to a single 6.25 mg dose of ochratoxin A (OTA) by oral gavage to 15 months male rats was progressive loss of weight during the following four days. Lost weight was restored within one month and animals had a normal life-span without OTA-related terminal disease. Decline in plasma OTA concentration only commenced four days after dosing, while urinary excretion of OTA and ochratoxin alpha was ongoing. During a temporary period of acute polyuria, a linear relationship between urine output and creatinine concentration persisted. Elimination of other common urinary solutes relative to creatinine was generally maintained during the polyuria phase, except that phosphate excretion increased temporarily. ^1^H NMR metabolomic analysis of urine revealed a progressive cyclic shift in the group principal components data cluster from before dosing, throughout the acute insult phase, and returning almost completely to normality when tested six months later. Renal insult by OTA was detected by ^1^H NMR within a day of dosing, as the most sensitive early indicator. Notable biomarkers were trimethylamine *N*-oxide and an aromatic urinary profile dominated by phenylacetylglycine. Tolerance of such a large acute insult by OTA, assessed by rat natural lifetime outcomes, adds a new dimension to toxicology of this xenobiotic.

## 1. Introduction

Many acute and chronic dose-response studies have been made on ochratoxicosis in rats in past decades on account of the general and nephro-toxicity of ochratoxin A [1,2]. More recent advance in analytical methodology has facilitated more complex evaluations spanning exposure to ochratoxin A (OTA) over one or more days, or several weeks at doses that are generally sub-clinical in effect or are not markedly toxic [3,4,5]. In any case these exposures were several orders of magnitude greater than is the common extent of human dietary exposure, typically for example via agricultural cereals and their products and tropical spices, and occasionally via coffee and cocoa. Indirect exposure via pig kidney or liver can also occur. The possibility that the renal carcinogenicity of OTA in the rat could be a model for human risk of urinary tract malignancy has been a significant factor in food safety legislation. Whereas the NTP study [6] defined a dose range which was carcinogenic during lifetime exposure, the relative roles of exposure period and cumulative amount of carcinogen were unknown. Cumulative carcinogen dose has even been found to be a better exposure metric than daily dose [7]. The context of recent EU-sponsored studies on renal carcinogenesis by OTA [8,9] has provided experimental opportunity also to demonstrate an obligatory period of 6–9 months exposure, only in the first year of male rat life [10], but also for the first time to follow outcomes after a single large oral dose of OTA for the rest of natural life. This is an exploratory step towards assessing a cumulative OTA exposure for rat renal carcinogenesis. Because of the long plasma half-life of OTA, a long cumulative period of renal exposure to the toxin might be expected from a single large dose, but such had not previously been explored. The findings, presently described, show the outcomes concerning pharmacokinetics and renal function, evolution of the urinary metabolomic profile through the immediate post-dosing phase and observations at necropsy. Since a quite marked, though tolerable, toxic impact was desired, mature adult males of the Fischer strain have been used instead of the young growing animals commonly used in toxicology studies.

## 2. Materials and Methods

### 2.1. Animal Experiment

Three male Fischer rats, 15 months old (445, 486 and 517 g) had been caged together since supplied from B and K Universal Ltd., Grimston, Hull, and housed contemporary with the control group as previously described [8]. They were fed maintenance (14% protein) rat diet (Special Diet Services, Witham, Essex), and water, *ad libitum.*

OTA (60 mg) was dissolved in ethanol (1 mL) and diluted with 4% sodium bicarbonate solution (4 mL). Rats were given 0.5 mL of the solution by oral gavage, in which therefore ethanol (100 µL) was also given. The solution was analysed quantitatively by HPLC with diode array detection [11] and found to contain 12.5 mg OTA/mL and 0.15 mg OTB/mL. Therefore the OTA dose administered to each animal was 6.25 mg. This was only a little above half of the published acute oral LD 50 dose for male rats [12] and was not intended to cause severe morbidity. All experimentation was conducted according to UK Home Office licence requirements.

### 2.2. Histopathology

Standard wax-embedded blocks were prepared from kidneys and all tumour tissues, and sections (3–4 µm) were stained with haematoxylin and eosin in the Breast Pathology laboratory, Guy’s Hospital, London.

### 2.3. Urinalysis

Automated urinalysis for creatinine, protein, calcium, sodium, potassium, urate, urea and phosphate was performed in the Chemical Pathology Laboratory at St. Mary’s Hospital, Paddington, London, using an Olympus AU640 instrument with methodology described in [13]. 

### 2.4. Analysis of Ochratoxin A in Rat Plasma

Measurement of the concentration of OTA in rat plasma was contracted to the standardised and validated protocol at the Central Science Laboratory, York, UK. The limit of measurable detection was <0.1 µg/mL, which takes into account the considerable dilution of most of the samples to be in the usual range for HPLC analysis. Samples came from only 200 µL of blood, withdrawn from a tail vein under general anaesthesia, from which plasma was immediately separated (eppendorf centrifuge) and stored at −20 °C. The analytical protocol, immuno-affinity cartridge clean-up followed by HPLC with fluorescence detection, was as previously described [14,15].

### 2.5. Measurement of Ochratoxin A in Rat Urine

The quantity of OTA present in each urine sample was assessed by addition of a known quantity of authentic standard, as given to the rats and used elsewhere [3], with measurement via ultra-performance liquid chromatography-mass spectrometry (Waters Acquity and LCTpremierMS) with a stepwise linear gradient. A 20 µL sample was injected onto a C_18_ BEH Acquity column and separated using a mobile phase of water plus 0.1% formic acid (solvent A) and acetonitrile plus 0.1% formic acid (solvent B). The LC run was isocratic up to 1.5 min with mobile phase of 0.5% B, followed by gradient elution (column temperature was 40 °C). The gradient was 1.5–5 min, 0.5–30% B; 5–15 min, 30–70% B; 15–18 min, 70–99.5% B; 18–20 min, 99.5–0.5% B. At all stages the flow rate was 0.5 mL/min and the data were acquired in positive mode. Measurement of the integral of the OTA peak was made of the sample before and after spiking with a known concentration of standard and the total urinary concentration was calculated from relative intensity of the m/z 404.089 parent ion eluting at 9.3 min. 

### 2.6. Measurement of Ochratoxin Alpha in Rat Urine

The quantity of ochratoxin alpha present in the 8–25 h urine sample from Rat B was assessed by addition of a known quantity of authentic standard (courtesy of H. Zepnik, University of Wuertzburg) with measurement via high-performance liquid chromatography-mass spectrometry (Waters Aliance 2795 and QToFmicro). A 10 L sample was injected onto a C_18_ Symmetry column and separated using a mobile phase of water plus 0.1% formic acid (solvent A) and acetonitrile plus 0.1% formic acid (solvent B). The LC run was isocratic up to 0.5 min with mobile phase of 100% A, followed by gradient elution (column temperature was 40 °C). The gradient was 0.5–4 min, 0–20% B; 4–8 min, 20–95% B; 8–9 min, 95–95% B; 9–9.1 min, 95–0% B. At all stages the flow rate was 0.6 mL/min and the data were acquired in negative mode. The total urinary concentration was calculated from relative intensity of the m/z 255.006 parent ion eluting at 5.8 min.

### 2.7. ^1^H NMR Spectroscopic Analysis of Whole Rat Urine

Each sample (400 µL) was mixed with 200 µL of phosphate buffer (0.2 M NaH_2_PO_4_: 0.2 M Na_2_HPO_4_ (19:81), pH 7.4). Aliquots of the resulting mixture (500 µL) were placed in 5 mm NMR tubes to which 50 µL of a solution of TSP in D_2_O was added (final concentration, 1 mM). The D_2_O plus TSP addition provided both a chemical shift reference (δ0.0) and a field frequency lock signal. One-dimensional (1D) ^1^H NMR spectra were acquired at 600.13 MHz on a Bruker DRX-600 spectrometer using a standard pre-saturation pulse sequence for water suppression with solvent irradiation in the relaxation delay (3 s) and the mixing time (100 ms). NMR spectra were acquired using 256 scans into 64 k points with a spectral width of 9615 Hz, an acquisition time of 3.4 s, and a total pulse recycle delay of 6.25 s. The FIDs were multiplied by an exponential function corresponding to a 0.3 Hz line broadening prior to Fourier transformation (FT). All data were phased and baseline corrected manually in TopSpin (version 2.5, Bruker GmbH, Germany).

### 2.8. Multivariate Statistical Analysis of ^1^H NMR Spectral Data

All NMR spectra were processed for multivariate statistical analysis using the AMIX software (version 3.9.7, Bruker GmbH, Germany). The spectra were normalised to the total integral (excluding water, urea and TSP) and data reduced to a series of 0.02 ppm integrated regions for analysis. The reduced dataset was pre-processed (pareto scaling) and analysed via Principal Components Analysis (PCA) and Partial Least Squares-Discriminant Analysis (PLS-DA) using the SIMCA-P+ software package (version 11.5, Umetrics, Umea, Sweden). Initial data analysis focused on comparison of the data from all samples to determine the time-periods of effect and recovery. Subsequently individual comparisons of the loadings data from the pre-dose samples were made with each of the post-dose sample groups to determine the specific endogenous metabolites differing at each time-period. Assignment of the NMR spectral resonances highlighted from this analysis were made based on SBASE2.0 (Bruker GmbH, Germany) and online resources [16].

## 3. Results

### 3.1. The Acute Toxicity Phase

**Figure 1:**
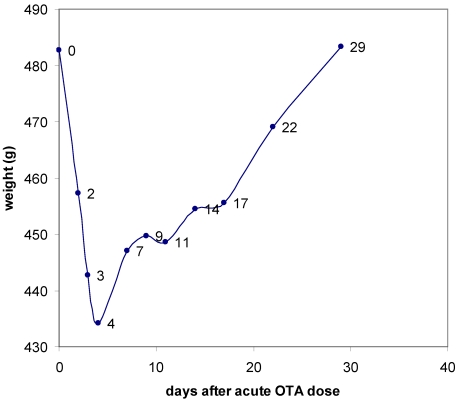
The course of change in mean rat weight during the month after an acute 6.25 mg OTA dose, showing sharp recovery after four days of weight loss.

After administration of OTA rats lost ~10% of body mass during the next 3–4 days (Figure 1), but after 4 days the lost mass was gradually recovered during the following month. Since rats were in metabolism cages for 18 h on days 1, 3 and 4 and were anaesthetised briefly for blood sampling six times during the period of mass loss, albeit always with access to feed and water *ad libitum*, it is not possible to distinguish between these experimental factors and any other direct toxicity-based inappetence in causing the loss.

**Figure 2:**
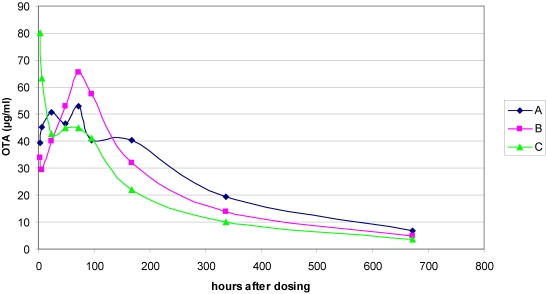
Plasma OTA concentration at intervals for individuals during the month following a 6.25 mg OTA dose to three adult male Fischer rats.

Absorption efficiency of intestinal OTA was indicated by achievement of maximum, or near maximum, plasma concentration measured 7 h after dosing. Concentration values in the 50 µg/mL range were maintained in all animals during the following 4 days (Supplementary data, Figure 1) and the pattern over a month is shown in Figure 2. Assuming plasma volume represented ~4% of body mass in these mature males, OTA in the total vascular compartment accounted for only ~10–15% of that administered. Six months later, OTA in the urine output during 18 h overnight was measured at 5.2 µg (±0.8 µg), demonstrating that the toxin was still in the blood in amounts greater than the trace level that might be acquired from commercial rat diet. In the intervening period, the pattern of plasma OTA concentration decline was similar in all animals (Figure 2). Log mean values at the various time points plotted against time (Supplementary data, Figure 2) show the decline more clearly, and its linear shape which fits a plasma half-life of ~9 days that is close to that measured in animals receiving long-term dietary exposure to OTA [15].

Polyuria became evident on days 3 and 4 (Table 1) and was the only overt indicator of toxicity. Urinalysis showed that creatinine concentration was correlated with polyuria. Plotting urine output against creatinine concentration showed a consistent linear relationship in all animals (Supplementary data, Figure 3) indicating an OTA effect on water retention in the distal part of the nephrons. Expression of concentration of other urinary parameters relative to excretion of one mmole of creatinine (Table 1) revealed no consistent pattern of marked changes for calcium, urate, protein, sodium or potassium. However, increase in phosphate excretion through the four days post-dosing may be a significant temporary change, but phosphate excretion had reverted to the pre-dosing status when measured again six months later. Urinalysis findings one year after dosing in one rat (rat A, Table 1) are notable only for much elevated excretion of calcium, protein and phosphate in this ageing (21 month old, but not leukaemic) animal. 

**Table 1:** Urinalysis data (±SD) for 18 h collections from rats at intervals after gavage dosing of 6.25 mg OTA. Units; volume (mL), creatinine (mmol/L), and other parameters (mmol/mmol creatinine).

	**Volumes**	**Creatinine**	**Calcium**	**Urate**	**Protein**	**Na**	**K**	**PO_4_**	**Urea**
Pre-OTA	7.5 (2.60)	10.63 (2.65)	0.31 (0.02)	0.12 (0.04)	0.40 (0.16)	7.54 (3.18)	19.45 (4.62)	2.28 (0.77)	87.39 (6.79)
8–25 h	7.17 (1.04)	9.18 (0.98)	0.33 (0.09)	0.2 (0.05)	0.39 (0.16)	12.32 (4.77)	12.83 (2.03)	3.55 (1.40)	77.27 (0.67)
55–74 h	15.8 (10.37)	6.50 (3.94)	0.18 (0.11)	0.2 (0.15)	0.42 (0.12)	11.67 (2.24)	12.66 (0.82)	5.56 (0.68)	60.14 (2.02)
79–88 h	30.7 (2.87)	2.29 (0.13)	0.13 (0.09)	0.1 (0.02)	0.63 (0.07)	7.03 (1.79)	18.76 (2.05)	4.41 (1.02)	58.00 (1.82)
6 months	7.97 (1.56)	10.55 (1.96)	0.27 (0.11)	0.17 (0.02)	0.37 (0.23)	3.39 (1.20)	14.93 (1.46)	2.64 (0.40)	61.44 (1.64)
12 months	14.5	4.66	1.28	0.34	3.04	10.33	22.27	6.19	52.56

**Figure 3:**
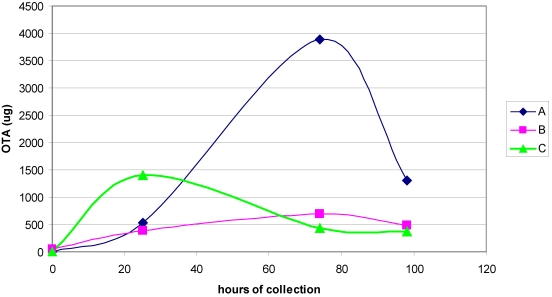
Urinary 18-h output of OTA before and during the four days after a 6.25 mg oral dose to three adult male rats.

Measurement of OTA excreted in urine during 18 h periods at intervals early in the experiment (Figure 3) showed significant output during the first day for all animals. Thereafter on days 3 and 4 a similar situation generally prevailed except for a higher value on day 3 for one rat. While the analytical method was not officially validated for quantitative purposes, its reliance on ion current from a specific OTA MS fragment at least makes quantification relative to an OTA standard meaningful within the group of urines analysed. 

Sensitive and specific urinalysis for ochratoxin alpha, with limit of detection at ~300 fg/µL enabled measurement in the urine of rat B excreted during the 8–25 h post-dosing period as 10.2 µg, representing 0.3% of the OTA given.

### 3.2. Survival and Necropsy

Survival for 23, 25 and 27 months (Supplementary data, Figure 4), is within the normal lifetime range for Fischer male rats. Rat A was euthanized somewhat prematurely due to a facial abscess, but there were no obvious internal abnormalities and no tumours were found in kidneys. Rat B developed leukaemia, confirmed by the splenomegaly (16 g) and mottled liver found at necropsy, but there was no renal histopathological change atypical for an old rat. Rat C was also leukaemic (spleen 16.7 g, spongy liver); kidneys were not abnormal in size or colour and contained no tumour, but testes had atrophied.

### 3.3. Metabolomics

**Figure 4:**
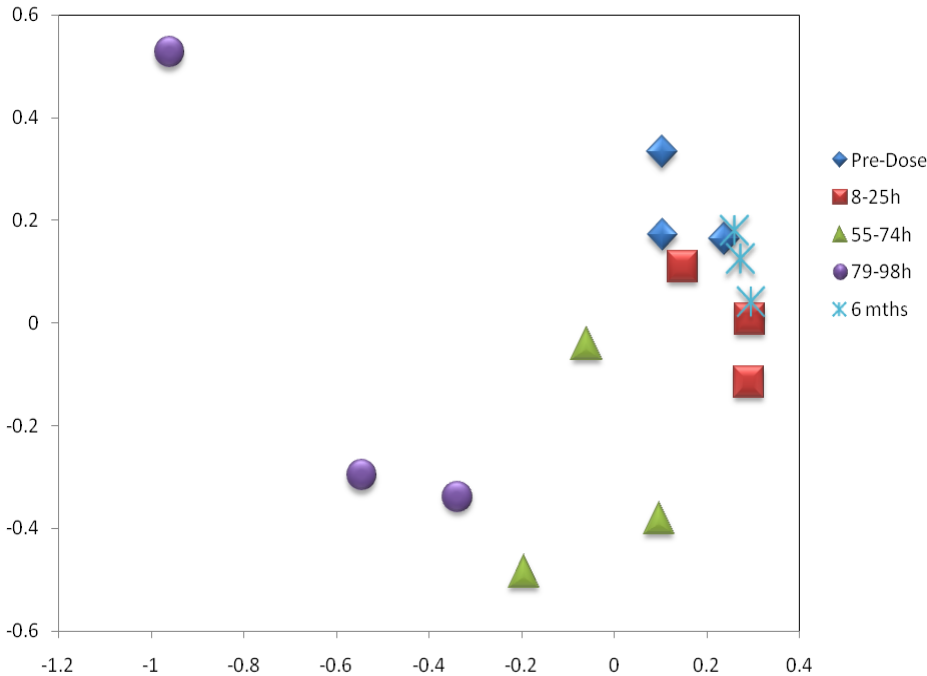
PC1 *vs*. PC2 scores plot of the NMR data from the three rats over the time-course of the study.

Principal component analysis data, from comparative analysis of ^1^H NMR spectra, is plotted graphically for individual animals for the five sampling stages including pre-dose and 6 months post-dose (Figure 4). Clustering of data from the three rats at the various stages progressed through distinct distance shifts along a cyclic pattern which eventually, after 6 months, had returned to the pre-dose status, thus demonstrating both kinetics of the toxic insult and its apparently-complete eventual resolution.

**Figure 5:**
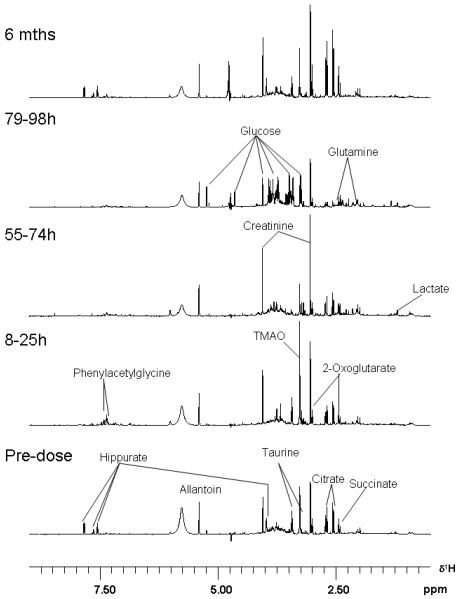
Stack plot of NMR spectra for rat B, pre-dose, after one, three and four days, and after six months. Spectra are representative of the three rats.

Examples of spectra showing the different patterns of urine components through the toxic insult, and later to recovery, are given as a stack plot in Figure 5, representing all samples from Rat A, which exhibited the least severe polyuria during the 79–98 h collection period. The spectra are normalised to the total integral of the displayed region. Notably, all three rats had a similar pattern of weight loss through the four days after dosing, illustrated in the body weight data in Figure 1. The stacked data in Figure 5 are therefore a conservative illustration for the whole group. The compounds which showed notable change in urine through the study, as identified from the statistical analysis, are listed in Table 2. The small amount of ethanol in the OTA dose vehicle was undetectable.

Specifically and chronologically, in the 8–25 h post-dose period hippurate virtually disappeared as a urine component and only reappeared in the 6 month sample. Phenylacetylglycine was a significant temporary component, but had become virtually absent three days later. Trimethylamine-*N*-oxide (TMAO) was also a prominent new component, recognised as additive to a taurine resonance, but then could not be detected by 79–98 h. New signals from an unassigned metabolite were also observed at δ7.47 (t) and δ7.34 (t).

Analysis of the loadings influencing differentiation of the predose urine samples from those collected at 55–74 h indicated that the affects observed post-dose were dominated by an elevation in citrate, 2-oxoglutarate, succinate, allantoin and creatinine. Changes evident as decreased excretion were taurine, trimethylamine-*N*-oxide and hippurate, but by 79–98 h further diminution of their resonances was observed.

The temporal shift from the pre-dose and 8–25 h post-dose periods through to the 79–98 h period was due to a glycosuria, aminoaciduria (principally concerning glutamine) and lactic aciduria coupled with reduced hippurate and citrate concentration. Reduced signal intensities were consistent with marked polyuria in all animals (Figure 5).

**Table 2:** Endogenous metabolites affected by administration of Ochratoxin A.

**Metabolite**	**Time-Period of Change**	**Delta**
Hippurate	8–25	decrease
Phenylacetylglycine	8–25	increase
TMAO	8–25	increase
Citrate	55–74	increase
2-Oxoglutarate	55–74	increase
Succinate	55–74	increase
Allantoin	55–74	increase
Creatinine	55–74	increase
Taurine	55–74	decrease
TMAO	55–74	decrease
Hippurate	55–74	decrease
Glucose	79–98 h	increase
Glutamine	79–98 h	increase
Lactate	79–98 h	increase
Hippurate	79–98 h	decrease
Citrate	79–98 h	decrease

## 4. Discussion

Our study uniquely demonstrates a remarkable recovery in renal function after a relatively large acute dose of OTA, implying that the insult had not caused profound structural damage and that renal carcinogenicity of the toxin operates via sub-clinical influences during the necessary exposure period of several months in the first year of life [10]. The eight day plasma half-life of OTA in these Fischer rats, being shorter than the ten day value measured when chronic OTA exposure ceases [15], at least indicates that no significant perturbation of renal function or long-term health problem persisted beyond the four days of post-dose morbidity. Ultimate tolerance of the maximum OTA that might safely be given was evident as rats regained their lost weight (presumably via lipid reserves). From previous experience [17], the present dose would not have been consumed if given in feed, implying an intrinsic effect of high dose of OTA on appetite. Animals lived into the normal life span for the Fischer strain and presented no unexpected pathology at necropsy other than is typical in aged rats. Notably no macroscopic or microscopic neoplasms were evident in kidney or in testis. Polyuria has also been reported after 7 and 21 days of OTA exposure (500 µg/kg/day) [18]. The single OTA dose here represented an amount equivalent to the cumulative daily intake that would be necessary during ~250 days to cause at least a 5% incidence of renal cell carcinoma [9]. Thus in the present experiment an acute OTA dose of ~40 mg might have been necessary to represent the cumulative requirement for a high incidence of renal carcinoma if delivered during a similarly protracted period. Predictably, that acute dose would have been lethal. A single OTA dose of 1 mg to Fischer rats caused a simple pattern of covalent DNA adducts in kidney [19], confirming exposure to the carcinogen, but that finding alone could not logically predict carcinogenesis without corresponding evidence that the long obligatory period of repeated insults with the toxin had occurred. Further, the present rats were exposed to OTA only in their second year of life, during which Fischer male rats have demonstrated much less response to carcinogenicity of OTA [20]. However, at least renal adenoma could have been expected from exposure in second year. Nevertheless, for at least a month, OTA was circulating in the present rats at a concentration in plasma above that which is potentially able to cause rat renal tumours if continued for about 9 months [10,15]. Thus the present findings can be a step towards experimental verification of the extent to which cumulative OTA exposure could be a predictor of cancer [21] in a rat model.

The maximum amount of OTA circulating in the vascular compartment during the first day in the present experiment (~1 mg) accounts for only ~15–20% of the given dose, but this was contemporary with typical renal excretory function as indicated by detection of both OTA and ochratoxin alpha in the urine. In [22] ochratoxin alpha was measured as the principal urinary metabolite of OTA, eventually accounting for about 25% of the mycotoxin administered parenterally once, and therefore the present finding confirmed typical OTA metabolism occurring through the period of uptake. It was also established [4] that OTA excretion in urine increased steadily over two weeks of dosing, though with dosing only on five consecutive days each week, in a dose-dependent and proportionate way. However, an interesting concomitant pattern in two separate cycles of excretion of ochratoxin alpha [4] indicated a profound change in OTA metabolism kinetics in kidney as soon as the first dosing period ended, while OTA concentration in the plasma circulating through the organ was well maintained.

The present experiment extends the direct relationship in Fischer rats between single dose amount, e.g., 0.5 mg/kg body weight [3,23,24] and maximum plasma concentration (~2 µg/mL), by showing here that a 25-fold increase in dose gave a mean plasma concentration of ~50 µg/mL. Also, the present single OTA dose was ~60-fold greater than that of the “lifetime” high-dose regimen in the classic NTP study [6], and allowed plasma OTA concentration to remain for ~35 weeks above that(1–2 µg/mL) associated with causing a very low incidence of renal cancer when given throughout life [9].

**Figure 6:**
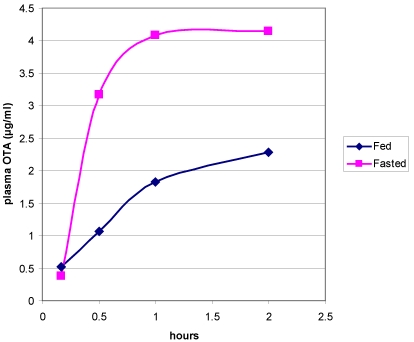
Expansion of mean plasma OTA measurements during the two hours post-dose in fed and fasted male Fischer rats showing the contrasting rates of uptake (after Vettorazzi *et al.* 2010).

Notably in [24], quicker OTA uptake was shown in fasted adult males than fed males in the first two hours, and achieved double the plasma concentration (Figure 6). Presumably OTA had been mostly bound to plasma proteins in both situations. It is no surprise that this could not easily be explained when so little is known about the kinetic fate of OTA molecules after entering the blood compartment and being subject to competition of protein binding of the 15–20% minority, and distribution of the remaining majority throughout the body. Far from just being unexplained, the robust analytical findings [24] could be reflecting the profound qualitative and quantitative changes in plasma proteins through puberty in male rats, as evidenced at least by those that escape to become the major group of urinary proteins which are becoming of increasing biological interest [25].

In the study with male Fischer rats in early puberty [4], conducted with exactly the same OTA material as here (cited purity 99.9%), metabolomic urinary changes were detected after two weeks of gavage treatment with OTA (1 mg/kg), but became more apparent at twice that dose. The highest daily dose (2 mg/kg), six-fold less than used presently, impaired growth slightly, but caused progressively greater polyuria. Aromatic amino acids were recognised only in the day after the first dose. However, trimethylamine *N*-oxide was already strikingly apparent 2 h after the first dose, declining in intensity thereafter but still evident after two weeks of treatment. Trimethylamine *N*-oxide concentration decline was in spite of continued dosing, which elevated plasma OTA concentration ~50-fold during the period. Subsequently, it was confirmed [5] that, during 13 weeks of exposure, the low OTA doses as tested [4] did not cause excretion of trimethylamine *N*-oxide. It was suggested that this was a specific toxicological feature of OTA. However, the present excretion of trimethylamine *N*-oxide just in the first day after dosing, in spite of very high plasma OTA concentration persisting thereafter, implies that this biomarker may occur in kidney as a shock response to OTA. Occurrence of trimethylaminuria was regarded [26] as a defect in detoxication, whereas it was originally proposed [27] that the metabolite was an indicator, at least also in Fischer rats, of site-specific renal papillary injury concomitant with polyuria, as can be caused by propylene imine. If OTA renal toxicology elicits a similar response at first exposure, it seems to happen 2–3 days before polyuria becomes manifest. It is not clear whether polyuria is due to direct interaction with the toxin in collecting ducts or through more distant adrenal or pituitary influence.

Metabolomic change in the first day following the OTA dose characterised by phenylacetylglycine excretion is reminiscent of response of germ-free rats to transfer to a conventional housing environment [28]. Although urinary tricarboxylic acid cycle intermediates have been shown to be affected by reduced food consumption [29], this is normally manifest as a reduction. The compounds which were elevated here have also been similarly elevated in animals that exhibit an idiopathic susceptibility to developing a renal toxic response following cisplatin administration [27]. As with OTA, cisplatin’s target for histopathological change is the *pars recta* region of nephrons. 

Overall in the acute phase, the elevated urinary citrate and hippurate most probably reflected an induced mild anorexia resulting in lower availability of TCA intermediates (hence reduced urinary citrate) and a stressor to the intestinal gut microflora (the principal contributor to urinary hippurate). The increase in markers of tubular damage in the kidney, assessed from considerable background experience of ^1^H NMR spectroscopy-based metabolomic assessment of toxicity [30,31], indicated that by the 79–98 h post-dose stage an adverse event was manifest in the organ. However, weight gain in all animals resumed a day later (Figure 1) implying both improved appetite and recovery of efficient medullary regulation of urinary output volume.

The present findings challenge current understanding about distribution and transport mechanisms, and raise questions about pharmacokinetics of OTA. OTA given via a jugular vein followed kinetics of immediately–declining plasma values in mature female Sprague-Dawley rats [32], showing a 4-day half-life after injecting 100 µg in aqueous vehicle. This route avoided the complexities of intestinal uptake and focused just on the rate of elimination. OTA (~125 µg) was given in corn oil by oral gavage to Fischer males [3]; the toxin had a longer plasma half life (9 days) in this rat strain. More recently [20], repetitive dosing in mature adult males took a month to reach maximum plasma concentration. Use of the ‘five-days-a-week oral gavage in an oil vehicle’ protocol of the NTP study [6] during two weeks [4], followed a similar pattern in young males.

Fischer rats have also been used [23,24] to focus on intestinal absorption in fed and fasted rats after single oral gavage dose of ~100 µg OTA in aqueous vehicle. Decline in plasma OTA concentration was already evident within one day of dosing. Notably also, gender difference in pharmacokinetics was found, but only in 15-week fed rats in which males achieved the significantly lower CMAX_obs_ value initially. The present protocol resembled the findings in [23] but with a greatly increased (25-fold) dose and a design which studied all animals thereafter for life. Decline in plasma OTA concentration was delayed for four days (Supplementary data, Figure 1) but thereafter, during the day 7 to day 28 period, the decline in log concentration of OTA in plasma followed a linear configuration when plotted against time (Figure 2), equating to a half-life value of eight days. It is assumed that there was considerable flux between the OTA initially distributed widely in tissues and that which entered the vascular compartment. The plasma concentration achieved during the first day demonstrated a proportional relationship with the oral dose over a wide dose range. Early establishment of excretion of OTA and its metabolite ochratoxin alpha allows conjecture about the mechanism of clearance from tissue into blood, the identity of plasma proteins with which the toxin binds competitively *in vivo*, the mechanism(s) and kinetics of competitive interactions, and the way(s) in which OTA that is mostly protein-bound in plasma is eliminated via nephrons. We perceive that what happens after an acute dose may not be the same as when plasma concentration stabilises after a month of continuous ingestion of OTA, otherwise plasma accumulation could hardly occur. Maintenance of a steady state concentration in plasma is very sensitive to cessation of OTA uptake [10,15], implying some metabolic adaptation during continuous exposure to OTA. The extent of rat plasma proteins to which OTA binds *in vivo* is unknown. Although binding to albumin has been modelled, no X-ray crystallographic data has apparently yet been presented; indeed, repeated attempts to obtain a crystal with a cloned human serum albumin at Imperial College London have unfortunately failed (with P. Zunszain and S. Curry). The extent of vascular involvement of OTA with male-rat urinary lipocalins, with short half-life in blood because they easily pass with glomerular filtrate, is unknown. From the literature, it is also difficult to have a clear picture of the dynamics of transport in kidney from blood to nephron epithelia and ultimately into urine. For example, a recent review [33] has excluded glomerular filtration of free OTA because of a classical reference to the strong binding of OTA to plasma protein. Consequently a complex scenario is proposed based on several *in vitro* studies on organic anion transporters, which offers explanation for accumulation of OTA in kidney. However, there was no recognition that protein binding of the phenylalanine derivative OTA is not irreversible and that the easiest exit of free toxin, maintained in blood at ~2% of plasma OTA by the principles of dissociation, is via glomerular fenestrations, as applies also to its analogue phenylalanine.

Further, the popular belief that OTA accumulates in rat kidney is mostly an illusion, since researchers persist in ignoring the OTA in the large vascular compartment which accounts for at least most of kidney OTA. Thus, in [3], although kidney was flushed with saline by hypodermic syringe inserted into the renal artery immediately after excision, it can be calculated that the OTA measured in blood plasma could have accounted for all OTA in kidney if only a residual 10% plasma component remained. Predictably, such flushing could not remove blood from peri-tubular vasculature without the benefit of blood pressure to keep vessels open [34]. Similar calculation can be made concerning other studies [4,35], based on the elegant computerised tomography [34]. The same could apply to OTA-DNA adducts in vascularised tissues, if the adducts also occur in blood.

During two weeks of gavage administration of OTA in corn oil to young Fischer male rats [4], mimicking the five days a week dosage regimen used in the NTP study [6], dose-related accumulation of OTA in plasma was recorded, and dose-related similar concentration in flash-frozen kidney and liver at the end of the experiment. Unfortunately, involvement of OTA in the proportionately large vascular compartment of both organs, which would have accounted for most or all of the organs’ OTA, was not recognised. For example, at 2 mg OTA/kg body weight, a plasma concentration of 40 µg/mL was achieved. OTA was measured in kidney as 3.2 µg/g, but plasma in the vascular compartment would have potentially contributed ~6 µg OTA/g tissue. Similarly when dosed at 1 mg OTA/kg, the ensuing plasma OTA content of 17 µg/mL would have contributed more than the OTA measured in tissues. Another study [17], also measuring OTA in plasma, kidney and liver, quotes plasma values after 7 and 21 days of daily dosing which account for all OTA in kidney and liver of Fischer male rats, assuming organ plasma composition only in the ranges 7.5–8% and 8.5–10%, respectively.

Such considerations might only be of academic significance if OTA and its toxicity did not command extensive scrutiny and legislation for food safety [1,2]. Yet, while no case of human ochratoxicosis has been established, there remains poor understanding of uptake and excretory mechanisms in the rat, which has been the principal experimental toxicological model for human kidney disease. The latter is typically focused towards the mainly idiopathic incidence of renal cell carcinoma as a global malignancy, and the regionally-specific Balkan endemic nephropathy with its characteristic bilateral renal atrophy and the urinary tract tumours that sometimes also occur. Even lifetime exposure to OTA does not cause renal atrophy or urinary tract tumours in rats. Fairly high intake of OTA over many months can cause carcinomas in renal parenchyma, but molecular work in progress points more towards analogy with the spontaneous renal tumours of the Eker rat than to humans. Thus for defining fundamentals in human disease aetiology and in making correct attribution of xenobiotic toxins in food safety it is regrettable that so little is known about OTA *in vivo*.

Further experimental definition of the OTA exposure criteria (e.g., dose *vs*. frequency *vs.* duration) necessary for causing the renal genetic damage necessary for carcinogenesis would greatly assist in moving toward a more enlightened and objective attitude to OTA as a mycotoxin of potential concern for human health. Greater understanding of male rat pharmacokinetics through the carcinogenetic period would assist in making a valid model for human risk.

## Supplementary Files

Supplementary File 1: Supplementary File (PDF, 14 KB)
