# Supplementary material for: 1H NMR Spectroscopy-Based Metabolomic Assessment of Uremic Toxicity, with Toxicological Outcomes, in Male Rats Following an Acute, Mid-Life Insult from Ochratoxin A
Source: Toxins (Basel). 2011 May 26;3(6):504–19. doi: 10.3390/toxins3060504 (PMC3202844; doi:10.3390/toxins3060504)

Supplementary Data

Supplementary Figure 1.

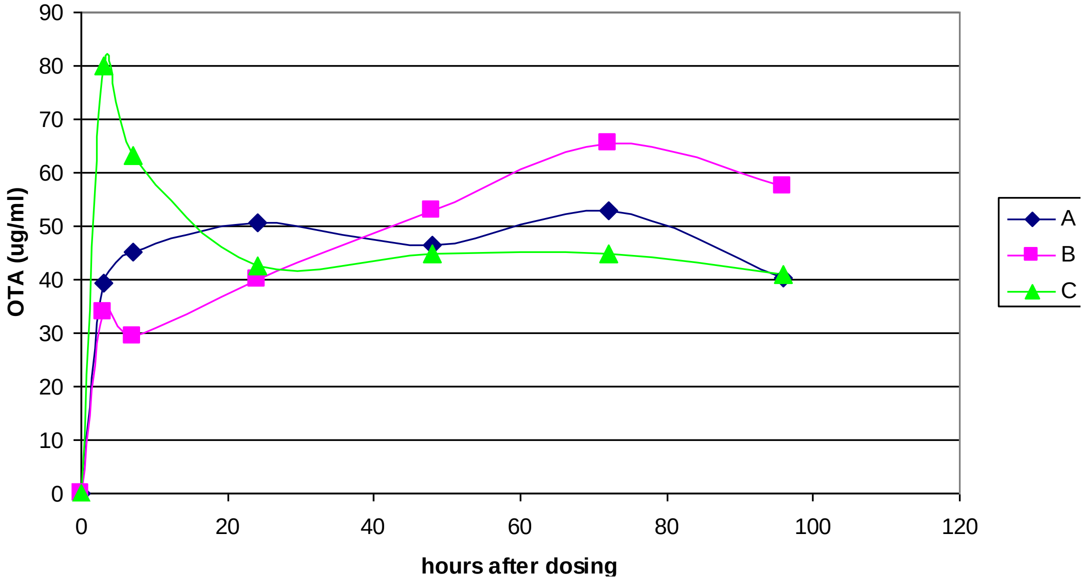

Supplementary Figure 2.

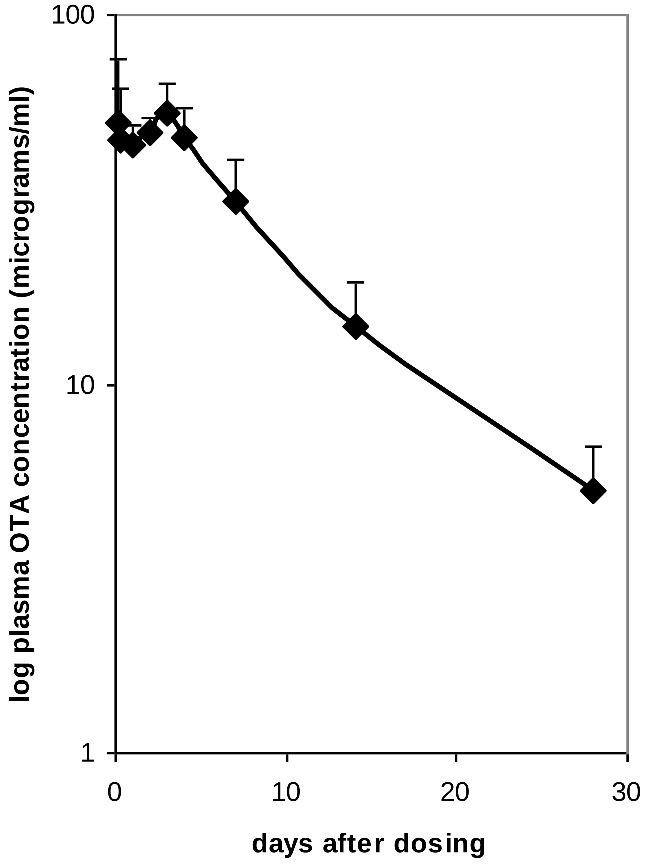

Supplementary Figure 3.

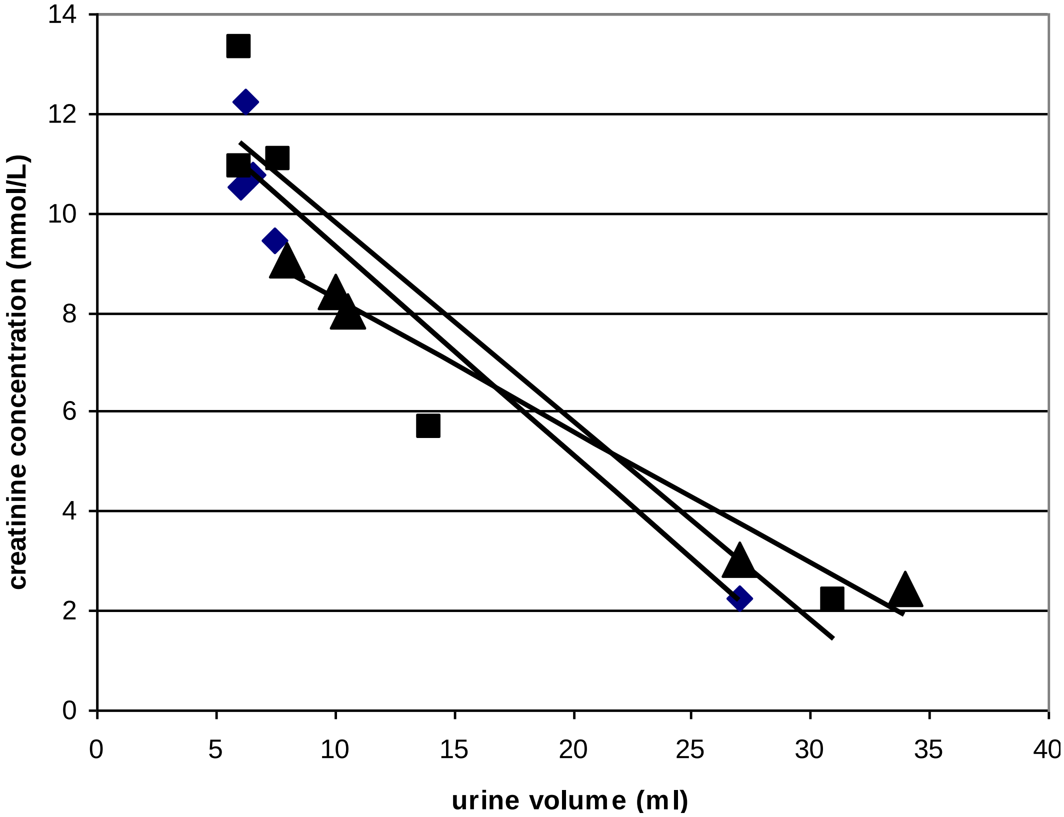

Supplementary Figure 4.

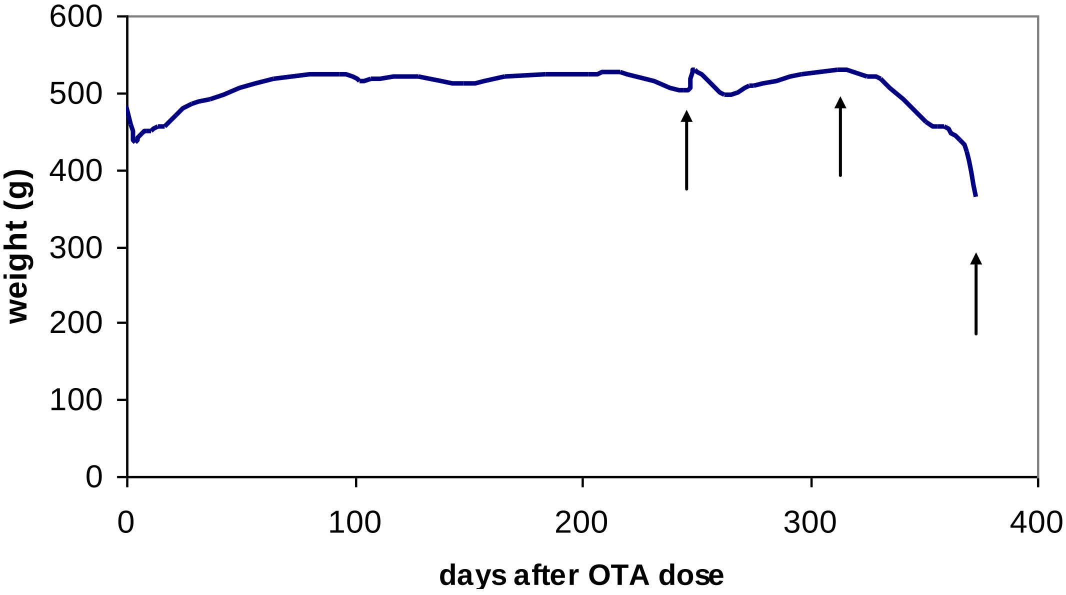

Supplement: Supplementary File 1: — Supplementary File (PDF, 14 KB) [file toxins-03-00504-s001.pdf]
